# Common Ribs of Inhibitory Synaptic Dysfunction in the Umbrella of Neurodevelopmental Disorders

**DOI:** 10.3389/fnmol.2018.00132

**Published:** 2018-04-24

**Authors:** Rachel Ali Rodriguez, Christina Joya, Rochelle M. Hines

**Affiliations:** Neuroscience Emphasis, Department of Psychology, University of Nevada, Las Vegas, Las Vegas, NV, United States

**Keywords:** neurodevelopment, GABA A receptor, autism spectrum disorders, Rett Syndrome (RTT), Angelman Syndrome, Dravet Syndrome, seizures, phasic and tonic inhibition

## Abstract

The term neurodevelopmental disorder (NDD) is an umbrella term used to group together a heterogeneous class of disorders characterized by disruption in cognition, emotion, and behavior, early in the developmental timescale. These disorders are heterogeneous, yet they share common behavioral symptomatology as well as overlapping genetic contributors, including proteins involved in the formation, specialization, and function of synaptic connections. Advances may arise from bridging the current knowledge on synapse related factors indicated from both human studies in NDD populations, and in animal models. Mounting evidence has shown a link to inhibitory synapse formation, specialization, and function among Autism, Angelman, Rett and Dravet syndromes. Inhibitory signaling is diverse, with numerous subtypes of inhibitory interneurons, phasic and tonic modes of inhibition, and the molecular and subcellular diversity of GABA_A_ receptors. We discuss common ribs of inhibitory synapse dysfunction in the umbrella of NDD, highlighting alterations in the developmental switch to inhibitory GABA, dysregulation of neuronal activity patterns by parvalbumin-positive interneurons, and impaired tonic inhibition. Increasing our basic understanding of inhibitory synapses, and their role in NDDs is likely to produce significant therapeutic advances in behavioral symptom alleviation for interrelated NDDs.

**Highlights:**

• Human studies and animal models need to be bridged in neurodevelopmental disorders
• Inhibitory signaling emerges as a common contributor to neurodevelopmental disorders
• Inhibitory signaling is diverse in mode, source, and target
• Systematic evaluation of inhibitory diversity is lacking in neurodevelopment
• Understanding of inhibitory signaling diversity will advance therapeutic strategies

## Introduction

Neurodevelopmental disorders (NDDs) are a broad class of disorders involving disruption in one or more domains including cognitive, emotional, motor, and/or behavioral function. NDDs affect up to 36.8% of children in low- and middle-income earning countries as evaluated in 2015 (Boivin et al., 2015; McCoy et al., 2016). Although NDDs have been the subject of extensive research, the common underlying mechanisms for behavioral and neurobiological symptomatology have yet to be systematically evaluated and compared among broad NDD subtypes. A basis for commonality among this complex and seemingly heterogeneous class of disorders lies in shared symptomatology (**Table 1**) and common genetic factors (**Table 2**). Further, genetic studies are beginning to indicate converging biological and phenotypic traits in complex disorders such as NDDs (Parikshak et al., 2016).

**Table 1:** Brief overview of symptom commonalities in NDDs.

Neurodevelopmental disorder	Autism-like characteristics	Intellectual disability	Epilepsy
Autism	Characteristic	Common	Prevalent
Angelman	Prevalent	Common	Prevalent
Rett	Prevalent	Common	Prevalent
Dravet	Rare	Common	Characteristic


**Table 2:** Summary of genes and proteins linked to synaptic function that are implicated, either directly through mutation or indirectly as secondary effects, in neurodevelopmental and associated disorders.

Gene human mouse	Encoded protein	Localization and/or function	Human populations	Mouse models
GABRA1 Gabra1	α1 subunit of the GABA_A_ receptor	Enriched at synaptic sites on dendrites and soma, relatively rapid decay	Epilepsy (Cossette et al., 2002) Dravet Syndrome (Carvill et al., 2014)	
GABRA2 Gabra2	α2 subunit of the GABA_A_ receptor	Enriched at synaptic sites on AIS, and early in development, relatively slow decay	Dravet Syndrome (Carvill et al., 2014)	Dravet Syndrome (Hawkins et al., 2016)
GABRA3 Gabra3	α3 subunit of the GABA_A_ receptor	Found in phasic receptors predominantly on the dendrites, high sensitivity to GABA	Epilepsy (Niturad et al., 2017)	Epilepsy (Niturad et al., 2017)
GABRA4 Gabra4	α4 subunit of the GABA_A_ receptor	Extrasynaptic localization (tonic), diazepam insensitive, high sensitivity to GABA	ASD (Ma et al., 2005; Sesarini et al., 2015)	
GABRA5 Gabra5	α5 subunit of the GABA_A_ receptor	Extrasynaptic localization (tonic), diazepam insensitive, high sensitivity to GABA	Angelman Syndrome (Pelc et al., 2008) ASD (Dup15q) (Park and Bolton, 2001; Kwasnicka-Crawford et al., 2007; Depienne et al., 2009)	Epilepsy (Vien et al., 2015)
GABRA6 Gabra6	α6 subunit of the GABA_A_ receptor	Extrasynaptic localization (tonic), diazepam insensitive, high sensitivity to GABA	Epilepsy (Hernandez et al., 2011)	
GABRB1 Gabra1	β1 subunit of the GABA_A_ receptor	Histamine, benzodiazepine, and anesthetic sensitive	ASD (Collins et al., 2006)	
GABRB2 Gabrb2	β2 subunit of the GABA_A_ receptor	Histamine, benzodiazepine, and anesthetic sensitive	Epilepsy (Srivastava et al., 2014) Schizophrenia (Lo et al., 2007)	
GABRB3 Gabrb3	β3 subunit of the GABA_A_ receptor		ASD (Dup15q) (Park and Bolton, 2001; Kwasnicka-Crawford et al., 2007; Depienne et al., 2009) Angelman Syndrome (Tanaka et al., 2012) Epilepsy (Tanaka et al., 2012)	Angelman Syndrome (Tanaka et al., 2012) Epilepsy (Tanaka et al., 2012; Vien et al., 2015)
GABRG2 Gabrg2	γ2 subunit of the GABA_A_ receptor	Most common subunit in synaptic receptors, relatively large channel conductance	Epilepsy (Baulac et al., 2001; Wallace et al., 2001) Dravet Syndrome (Huang et al., 2012)	
GABRG3 Gabrg3	γ3 subunit of the GABA_A_ receptor		ASD (Dup15q) (Park and Bolton, 2001; Kwasnicka-Crawford et al., 2007; Depienne et al., 2009) Angelman Syndrome (Pelc et al., 2008)	
GABRD Gabrd	δ subunit of the GABA_A_ receptor	Extrasynaptic localization (tonic), diazepam insensitive, high sensitivity to GABA	Epilepsy (Macdonald et al., 2012)	Epilepsy (Macdonald et al., 2012)
GPHN Gphn	Gephyrin	Scaffolding protein of inhibitory synapses, protein–cytoskeleton interaction	ASD (Chen et al., 2014)-rare	
ARHGEF9 Arhgef9	Rho guanine nucleotide exchange factor 9/collybistin	Scaffolding protein of inhibitory synapses	ASD (Chen et al., 2014)-rare Hyperekplexia (Tijssen and Rees, 1993; Kalscheuer et al., 2009; Alber et al., 2017) Intellectual disability (Kalscheuer et al., 2009; Long et al., 2015)	Hyperekplexia (Tijssen and Rees, 1993; Harvey et al., 2004)
NRXN1 Nrxn1	Neurexin1	Cell surface proteins involved in cell–cell interactions, export of secretory granules and modulation of signaling	ASD (Pizzarelli and Cherubini, 2011; Chen et al., 2014)	ASD (Pizzarelli and Cherubini, 2011; Grayton et al., 2013)
NRXN2 Nrxn2	Neurexin2			
NRXN3 Nrxn3	Neurexin3			
CNTNAP2 Cntnap2	Contactin associated protein-like 2 CSPR2	Role player in formation of domains for salutatory conduction of nervous impulses, part of neurexin family	ASD (Jacob, 2016; Rodenas-Cuadrado et al., 2016) Epilepsy (Jacob, 2016)	ASD (Peñagarikano et al., 2011)
NLGN1 Nlgn1	Neuroligin-1	Cell surface protein with role in synapse function via interaction with neurexin, localized at excitatory synapses	ASD (Pizzarelli and Cherubini, 2011; Chen et al., 2014)	ASD (Dahlhaus et al., 2010) Rett Syndrome (Pizzarelli and Cherubini, 2011) Fragile-X (Dahlhaus and El-Husseini, 2010)
NLGN2 Nlgn2	Neuroligin-2	Scaffolding protein that interacts with neurexins to aid in cell–cell interaction, localized at inhibitory synapses		ASD (Hines et al., 2008)
NLGN3 Nlgn3	Neuroligin-3	Cell surface protein with role in synapse function via interaction with neurexin, localized at excitatory and inhibitory synapses		ASD (Tabuchi et al., 2007; Jaramillo et al., 2014)
NLGN4 Nlgn4	Neuroligin-4	Cell surface protein involved in cell–cell interactions, localized at inhibitory synapses		ASD (Jamain et al., 2008)
CNTN Cntn	Contactins	Mediators of cell surface interactions during nervous system development	ASD (Bakkaloglu et al., 2008)	ASD (Banerjee et al., 2014)
SHANK2 Shank2	SH3 and multiple ankyrin repeat domains protein 2	Scaffolding protein of inhibitory synapses		ASD (Schmeisser et al., 2012; Banerjee et al., 2014)
SHANK3 Shank3	SH3 and multiple ankyrin repeat domains protein 3		ASD (Durand et al., 2007)	ASD (Banerjee et al., 2014; McGee et al., 2014)
MECP2 Mecp2	Methyl-CpG-binding protein 2	Chromosomal protein that binds to DNA that has been methylated	Rett Syndrome (Kozinetz et al., 1993; Amir et al., 1999; Banerjee et al., 2014) ASD (Tanaka et al., 2012)	Rett Syndrome (Chao et al., 2007; Medrihan et al., 2008)
UBE3A Ube3a	Ubiquitin-protein ligase E3A	Accepts ubiquitin from E2 and transfers to substrate	Angelman Syndrome (Jiang et al., 1998; Pelc et al., 2008) Epilepsy (Jiang et al., 1998)	


Autism spectrum disorder (ASD) is a prototypical NDD, and acts as a reference point for this class of disorders, with others often being described as possessing “autistic-like features.” Features of ASD include any range of impairments in social interaction, language development, and cognition, along with stereotyped behavior, and restricted interests (Rapin and Katzman, 1998). In addition to these core features, ASD is also frequently associated with epilepsy, impaired sensory processing, hyperactivity, and disrupted brain activity patterns as characterized by electroencephalogram (EEG) recordings (Rubenstein and Merzenich, 2003). These associated symptoms, in particular, hint that impaired inhibitory signaling is a contributor to ASD.

Other NDDs with similar symptoms to ASD, including Rett Syndrome (RS) and Angelman Syndrome (AS), have a clear genetic cause (**Table 2**). Specifically, RS is linked to the MECP2 gene (Banerjee et al., 2012), and AS linked to UBE3A and/or GABA_A_R subunit dysfunction through a loss of activity within the 15q11-13 locus (Malzac et al., 1998). The monogenetic nature of these disorders raises them as attractive targets to begin investigating NDDs. This is in contrast to ASD that is linked to an extremely heterogeneous and inconsistent genetic profile (Hu and Steinberg, 2009; Hu et al., 2009). Strikingly, RS and AS often share features of ASD, including the associated symptoms indicative of impaired inhibitory signaling. RS and AS also have a high incidence of epilepsy, with the vast majority of patients being affected by seizures (Fiumara et al., 2010; Vignoli et al., 2017). Comparing and contrasting the current knowledge on these disorders stands to provide information about the common neuropathological underpinnings of NDDs. In particular, examination of their association with epilepsy and other indications of impaired inhibitory control of principal cell firing, alongside an analysis of inhibitory synapse dysfunction could advance our understanding.

In typical development of the central nervous system, excitatory and inhibitory signals work in concert achieving regulated and orchestrated signaling patterns or oscillations. This tenet is the basis for a theory originally proposed by Rubenstein and Merzenich (2003), which suggests that NDDs are the result of an atypical “balance” between excitation and inhibition. An “imbalance” is proposed to increase the signal-to-noise ratio and decrease the efficiency of processing in afflicted individuals, leaving the cortex susceptible to seizures and other dysfunctional symptoms linked to hyperexcitability (Rubenstein and Merzenich, 2003). Mounting evidence is demonstrating that a number of genetic mutations can disrupt this balance, and in the case of NDDs, can tip the balance toward excitation at the expense of inhibition, contributing to multiple symptoms including a lower seizure threshold and a high incidence of epilepsy. Although the concept of an excitatory-inhibitory balance has been useful in understanding pathological and non-pathological brain states, it is likely an oversimplification and may not be sufficient to explain the immense heterogeneity and complex etiology of NDDs.

To advance from the excitatory-inhibitory balance hypothesis we need to understand synapses more fundamentally in the brain, particularly during development. We need both more detailed understanding of the developmental regulation of specific synapse types and implications for network activity, as well as more global understanding of the functional intersection between molecular and ionic states, synapse types, and network signaling during development. The current state of knowledge on inhibitory synapse development and regulation is limited compared to that of excitatory glutamatergic synapses. Furthering research in the field of inhibitory synapse development is essential to gaining insight into how this system impacts typical and atypical neurodevelopment. This review will examine the commonalities between seemingly heterogeneous NDDs, highlighting common genetic links and evidence of inhibitory synaptic dysfunction, in pursuit of a common rib in the umbrella of NDD.

## Inhibitory Signaling

**FIGURE 1:**
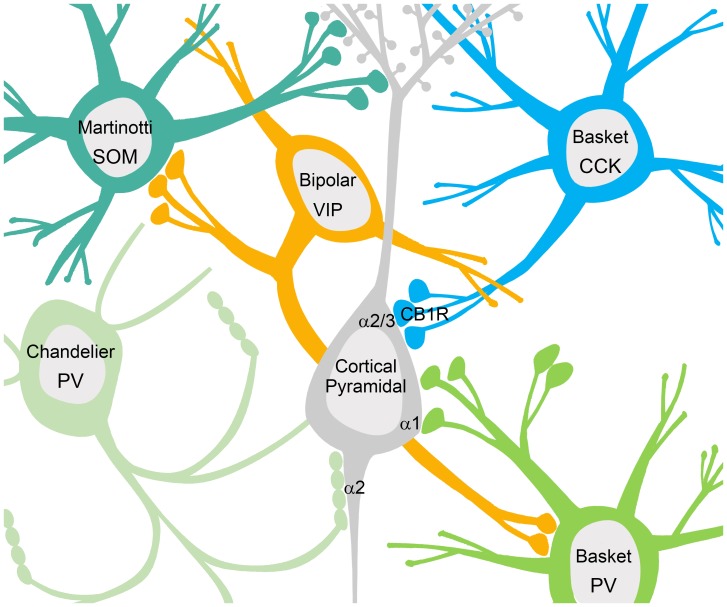
Interneurons contact multiple domains of their postsynaptic targets producing different forms of phasic inhibition. Somatostatin (SOM) positive Martinotti cells contact the dendritic domain to provide local inhibitory influences on excitatory inputs. Basket cells positive for CCK contact the soma where GABA_A_Rs containing the α2/3 subunits are enriched, while basket cells positive for parvalbumin (PV) contact the cell body at sites enriched with α1 containing GABA_A_Rs. PV positive chandelier cells project to the axon initial segment where GABA_A_Rs containing the α2 subunit are enriched. Somatic and axonal inhibition plays a role in coordinating neural activity patterns due to the proximity of the incoming graded potentials to the site of action potential generation. Disinhibitory interneurons innervate other interneurons providing net excitation, as seen with vasoactive intestinal polypeptide (VIP) positive bipolar cells.

Neuronal network signaling relies not only on excitation, but also heavily on inhibition to spatially and temporally pattern principal cell activity. Inhibition in the brain is governed by interneurons that synthesize and release the neurotransmitter γ-aminobutyric acid (GABA). A large body of work has documented and characterized 16 subtypes of interneurons in the hippocampus and/or cortex that possess unique morphologies, differential protein expression patterns, and specialized connectivity, physiological function, and functional impact within neuronal networks (**Figure 1**; Klausberger and Somogyi, 2008; Petilla Interneuron Nomenclature Group et al., 2008). A number of these interneuron types are relevant to the investigation of inhibitory signaling in NDD, due to their enrichment in cortical circuitry, and implication in patterning network activity. The majority of hippocampal and cortical interneurons express either the calcium binding protein Parvalbumin (PV), or the neuropeptide cholecystokinin (CCK) (Klausberger and Somogyi, 2008; Petilla Interneuron Nomenclature Group et al., 2008). PV-positive axo-axonic chandelier cells innervate the axon initial segment (AIS) of principal cells at or near the site of action potential generation, and can coordinate the activity of the up to 1,200 cells that they innervate (Li et al., 1992; Klausberger and Somogyi, 2008; Petilla Interneuron Nomenclature Group et al., 2008). PV-positive and CCK-positive basket cells innervate the soma and proximal dendrites of principal cells, and again play a role in coordinating activity patterns due to proximity to the AIS (Freund and Katona, 2007; Klausberger and Somogyi, 2008; Petilla Interneuron Nomenclature Group et al., 2008). In the cortex, Martinotti cells express the neuropeptide somatostatin (SOM) and innervate the dendrites of principal cells, providing local effects on incoming excitatory input (Rudy et al., 2011). In addition, hippocampal and cortical networks also include interneurons that innervate interneurons, thereby acting in a disinhibitory manner (**Figure 1**). One example in the cortex is a type of bipolar cell that is positive for the neuropeptide vasoactive intestinal polypeptide (VIP), which have been shown to target the majority of PV-positive interneurons (Dávid et al., 2007).

**FIGURE 2:**
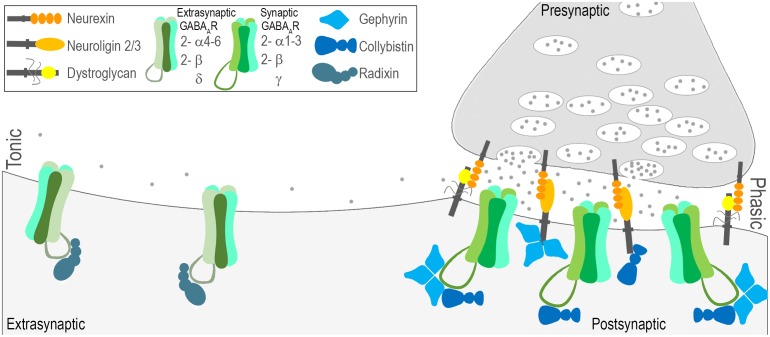
Molecular characterization of phasic and tonic modes of inhibition. Fast inhibitory synaptic transmission, also known as phasic inhibition, is governed by GABA_A_R’s with relatively low sensitivity to GABA densely clustered at sites opposing interneuron terminal boutons. Several general factors have been identified that play a role in establishing the contact between pre and post (neurexin-neuroligin2/3; neurexin-dystroglycan), and/or retaining receptors at these sites (gephyrin, collybistin). Phasic GABA_A_Rs are most commonly composed of 2-α1-3, 2-β, and a γ subunit and are sensitive to benzodiazepines. Tonic inhibition is governed by GABA_A_R’s that reside outside of synaptic sites, are highly sensitive to GABA, and are most commonly composed of 2-α4-6, 2-β, and a δ subunit. Much less is known about the mechanisms that localize extrasynaptic GABA_A_Rs, but radixin is implicated.

Once released by interneurons, GABA acts on ionotropic GABA type _A_ receptors (GABA_A_Rs; as well as metabotropic GABA type *_B_* receptors). GABA_A_Rs are heteropentamers gating a chloride channel, composed of combinations of subunits from seven families: α(1-6), β(1-3), γ(1-3), δ, ε, θ, π, and ρ(1-3) (Rudolph and Möhler, 2006). The genes and protein products for the most abundant CNS subunits are briefly described in **Table 2** and depicted in **Figure 2**. Subtypes of GABA_A_Rs composed of different subunit combinations have specific localization patterns and diverse functional impacts. In addition, GABA_A_Rs can be found at both synaptic and extrasynaptic sites mediating functionally distinct modes of inhibition (**Figure 2**). The most abundant subtypes of GABA_A_Rs are synaptic, sensitive to benzodiazepines, and composed of α1-3, β1-3, and γ2 subunits (Lüscher and Keller, 2004; Rudolph and Möhler, 2006; Jacob et al., 2008). In contrast, extrasynaptic, benzodiazepine insensitive receptors typically contain α4-6, β2/3, and δ subunits (Herd et al., 2013).

In addition to modulation by benzodiazepines, synaptic GABA_A_Rs are also the site of action for non-benzodiazepine Z-drugs (Dixon et al., 2015), and barbiturates, while extrasynaptic GABA_A_Rs are thought to be the site of action of intravenous and inhaled anesthetics (Chiara et al., 2016), anesthetic steroids, neurosteroids and other exogenous modulators such as ethanol (Brickley and Mody, 2012; Sigel and Steinmann, 2012; Olsen, 2015). In addition to clinically relevant ligands, there are also a number of preclinical or experimental ligands such as muscimol (synaptic), and gaboxadol (extrasynaptic), and the channel blocker picrotoxin (Sigel and Steinmann, 2012; Olsen, 2015). The diversity of localization and function, paired with the clear clinical and behavioral impact of pharmacological manipulation, make GABA_A_Rs an important target of study.

### Synaptic GABA_A_ Receptors – Phasic Inhibition

GABA_A_Rs located at synaptic contacts are activated in a transient or ‘phasic’ manner by a high concentration of GABA released from presynaptic vesicles (**Figure 2**, right). GABA released from the presynaptic neuron bind to GABA_A_Rs gating a channel permeable to chloride and bicarbonate ions at the postsynaptic membrane. Generally, this leads to a net inward flow of anions, and a hyperpolarizing postsynaptic response known as the inhibitory postsynaptic potential (IPSP). Defining features of this phasic mode of receptor activation are the rapid synchronous opening of a relatively small number of channels that are clustered at the synaptic junction and the short duration of the GABA transient to which the postsynaptic receptors are exposed. Phasic receptors enriched at synapses are most commonly characterized by two α(1-3), two β, and one γ subunit.

The clustering and accumulation of GABA_A_Rs at synaptic sites relies upon the interaction of multiple receptor-associated, adhesion, and scaffolding proteins diagramed in **Figure 2**, including gephyrin (Tretter et al., 2008, 2011; Mukherjee et al., 2011), collybistin (Saiepour et al., 2010), neuroligin 2 (Chih et al., 2005; Poulopoulos et al., 2009), neurexin (Zhang et al., 2010), and the dystroglycan, dystrophin–glycoprotein complex (Panzanelli et al., 2011; Früh et al., 2016). Despite a common pool of interacting proteins, heterogeneity exists in the complex mechanisms governing clustering of receptors containing different subunits. Composition is likely dependent upon the combination of interacting proteins involved, and their relative interaction affinities.

Inhibitory synapses that generate phasic inhibitory signals can be found on multiple compartments of the cell including the dendrites, axon, and cell body (**Figure 1**). For example, receptors containing α1 subunits are enriched at inhibitory synapses on the dendrites, those containing α2 subunits are preferentially enriched at inhibitory synapses on the AIS, and both α1 and α2 subunits can be found clustered at somatic inhibitory synapses (**Figure 1**; Nusser et al., 1996; Freund and Katona, 2007; Gao and Heldt, 2016). AIS synapses are unique to principal neurons in the hippocampus and cortex, and are thought to control principal cell activity patterns by influencing the site of action potential generation (Kuba et al., 2010; Wefelmeyer et al., 2015). As mentioned above, AIS synapses originate from chandelier cells, which are highly specialized, fast-spiking, PV-positive interneurons (Li et al., 1992; Klausberger and Somogyi, 2008; Petilla Interneuron Nomenclature Group et al., 2008). The cortex is also enriched with PV-positive, fast-spiking basket cells, and CCK positive basket cells. CCK positive basket cell terminals contact the soma of pyramidal cells where clusters of GABA_A_Rs containing the α2/3 subunits are enriched (Nyíri et al., 2001). The formation and maintenance of CCK terminals on the soma has recently been linked to dystroglycan, a protein responsible for many inherited forms of muscular dystrophy (**Figure 1**; Früh et al., 2016). Outside of the role for dystroglycan in CCK-basket cell contacts on the soma, detailed information about the mechanisms of other specialized subtypes of inhibitory synapses, such as those localized to the AIS is lacking.

In addition to localization and related impacts on function, subunit contents have also been shown to influence the physiological properties of GABA_A_Rs including the decay time of GABA-activated currents within single cells. Presence of a β2 subunit results in a decreased period of time in which the receptor chloride channel is open—leading to faster desensitization. In contrast, the γ2 subunit results in channels with larger conductance (Verdoorn et al., 1990). In addition, α1 subunit containing receptors show faster decay times compared to α2 containing receptors (Lavoie et al., 1997; Eyre et al., 2012).

### Extrasynaptic GABA_A_ Receptors – Tonic Inhibition

GABA_A_R activation can also take place in a less spatially and temporally restricted manner to generate a ‘tonic’ conductance (Nusser et al., 1998; Farrant and Nusser, 2005; Hines et al., 2012; Lee and Maguire, 2014). Low GABA concentrations in the extracellular space escaping from the synaptic cleft can activate GABA_A_Rs distant from sites of neurotransmitter release (**Figure 2**, left; Farrant and Nusser, 2005; Hines et al., 2012). The tonic mode of receptor activation has one straightforward outcome: a persistent increase in the cell’s input conductance. This increase in conductance affects the magnitude and duration of the voltage response to an injected current, and increases the decrement of voltage with distance (Farrant and Nusser, 2005; Bright and Smart, 2013). Tonic GABA_A_Rs exhibit a high sensitivity to GABA and show little desensitization, allowing them to detect and respond to ambient levels of GABA at extrasynaptic sites (Nusser and Mody, 2002; Farrant and Nusser, 2005; Hines et al., 2012). Tonic receptors are most commonly characterized by two α(4-6), two β and one δ subunit (**Figure 2**, left). GABA_A_Rs containing δ subunits tend to be purely extrasynaptic (Farrant and Nusser, 2005; Lee and Maguire, 2014). The α4 and α6 subunits are diazepam-insensitive receptors, as their inclusion results in decreased sensitivity to complete desensitization to benzodiazepine binding on the receptor (Whiting et al., 1995). While the complex mechanisms governing localization of GABA_A_Rs at synaptic sites have been somewhat clarified, very little is known about the mechanisms that are involved in regulation of extrasynaptic GABA_A_Rs. One of the few proteins implicated in regulation of extrasynaptic GABA_A_Rs is radixin, which has been shown to influence clustering of α5 containing receptors (**Figure 2**, left; Loebrich et al., 2006). Further research is needed to understand how these receptors are localized and regulated, and also to understand further how their action may be harnessed to control excitation.

### Chloride Ion Reversal and GABA Signaling

Another factor that can modulate the efficacy of GABAergic inhibition is the reversal potential for GABA_A_ (EGABA) receptor-mediated current and voltage responses. EGABA is controlled by the equilibrium potential for chloride (ECl) which in turn is regulated by the expression and function of cation-chloride cotransporters (CCCs; Rivera et al., 2005; Ben-Ari et al., 2007; Ben-Ari, 2014; Kaila et al., 2014). Two key members of the CCC family for controlling neuronal ECl are the potassium chloride cotransporter (KCC2) and the sodium potassium chloride cotransporter (NKCC1) which provide outwardly and inwardly directed chloride fluxes in neuronal cells, respectively (Ben-Ari et al., 2007; Hartmann and Nothwang, 2014; Doyon et al., 2016). In mature neurons, chloride is extruded by high levels of expression and function of KCC2, using the potassium gradient in order to maintain a low intracellular chloride concentration (Ben-Ari et al., 2007; Doyon et al., 2016). A low intracellular concentration of chloride is important to maintaining the driving force of chloride, such that when GABA binds, chloride will enter the cell through the pore of the GABA_A_R and hyperpolarize the cell (Ben-Ari, 2014; Kaila et al., 2014). Early in development, KCC2 is low in expression and activity in neurons leading to a high intracellular chloride concentration (Ben-Ari et al., 2007; Ben-Ari, 2014). High levels of chloride inside the neuron cause depolarization in response to GABA_A_R activation, and under specific conditions, excitatory GABAergic transmission (Rivera et al., 2005; Ben-Ari et al., 2007; Kaila et al., 2014). Excitatory GABA is proposed to play a trophic role in early development, with important effects on the cell cycle, cell migration, and primitive oscillatory patterns thought to influence network wiring (Represa and Ben-Ari, 2005). During development there is a caudal to rostral gradient in the increase of KCC2 expression, with the cortex showing continued increases in KCC2 expression well after birth (Kaila et al., 2014). Beyond the developmental state of high intracellular chloride and depolarizing GABA, it is now established that pathological states including epilepsy and trauma are also characterized by reduced KCC2 expression and high intracellular chloride (Kaila et al., 2014). Further understanding is required about the conditions for inducing change in the expression of KCC2, and also for the implications, that this has on circuit level function.

Understanding the distinctive functional contributions of GABAergic synapses on specific subcellular compartments, phasic and tonic forms of GABAergic signaling, as well as the impact of alterations in ECl will lead to greater ability to fine tune inhibitory signaling. An understanding that is currently lacking is of the neurodevelopmental processes that establish and maintain the structurally and functionally distinctive types of GABAergic synapses, as well as the roles for tonic inhibition throughout development. Further, we are continuing to understand the roles of excitatory GABA early in development. We need to evaluate perturbations in each of these specializations of GABAergic signaling in NDDs with a subunit specific, cell-domain specific level of detail, deciphering precisely which subtypes of GABA_A_Rs are affected, and what role the transition from excitatory to inhibitory GABA may play. In the following sections, we will describe the features of specific NDDs and highlight existing studies that have assessed GABAergic signaling in either NDD populations or disease models.

## Autism Spectrum Disorders

Autism spectrum disorder affects boys more commonly than girls, with a ratio between 3:1 and 4:1, with a prevalence of 1 in 54 boys (Coury et al., 2014; Loomes et al., 2017). The disorder is characterized by core symptomatology that includes compulsive or repetitive behaviors and social communication deficits (American Psychiatric Association, Diagnostic and Statistical Manual 5th Edn). These core symptoms are often accompanied by intellectual disability, sensory sensitivity, and seizures. Seizures (and/or epilepsy) occur in ∼30–60% of individuals with ASD (Rubenstein and Merzenich, 2003; Jacob, 2016). Historically, the relationship between ASD and abnormal EEG recordings was the first reason it was considered a brain disorder (Banerjee et al., 2014). This suggests that altered oscillatory activity is a prominent feature of ASD. The heritability of ASD has been studied in family and twin studies and shows a heritability rate of 70% (Tick et al., 2016). Accumulating genomic/genetic investigations indicate hundreds of genetic variants in the occurrence of ASD, including a range from common to rare variants (Krumm et al., 2014). Several of these identified genetic mutations producing autistic-like behaviors have been investigated in mouse models. Despite extensive research in human populations and mouse models, no unifying cause has been determined.

Multiple genetic contributors to ASD are synaptic proteins, and in particular can be classified as trans-synaptic adhesion molecules, or synaptic scaffolding proteins, or synaptic signaling proteins (**Table 2**). Specific genes implicated within these categories are discussed in detail below: adhesion molecules from the neurexin, neuroligin, and contactin families; synaptic scaffolding proteins from the shank family; and GABA_A_R subunit genes within the 15q11–13 locus.

### Adhesion Molecules: Neuroligin, Neurexin and Contactin Families of Proteins

The neurexin (NRXN/Nrxn), neuroligin (NLGN/Nlgn), and contactin (CNTN) families are cell adhesion molecules (CAMs; **Table 2**). In broad terms, CAMs are membrane proteins that extend into the extracellular space and facilitate physical interaction with other cells or with the extracellular matrix. NRXNs are enriched in the presynaptic compartment whereas NLGNs are enriched postsynaptically (Ichtchenko et al., 1995). The two families of proteins interact to form a transsynaptic bridge, linking pre to post, and are thought to be early contributors to synapse formation (Scheiffele et al., 2000; Levinson and El-Husseini, 2005). Within their respective compartments, these proteins also interact with components of the active zone and postsynaptic density, including components linked to neurotransmitter vesicle release, and neurotransmitter receptors and scaffolding proteins (Irie et al., 1997; Prange et al., 2004). The NRXN family includes three genes (NRXN1-3) that can be expressed via one of two promoters, termed α and β. Transcription via the α promoter yields a longer protein with six laminin/neurexin/sex hormone (LNS) binding domains, whereas transcription via the β promoter yields a shorter protein with a single LNS binding domain (Tabuchi and Südhof, 2002). NRXN proteins also contain extracellular epidermal growth factor (EGF) repeats and a PSD-95-Discs Large-zona occludens-1 (PDZ) binding motif. The NRXN family also includes the contactin associated protein (CNTNAP) genes that encode CASPR proteins that are structurally similar with LNS binding domains, EGF repeats, and a PDZ binding motif (Gennarini et al., 2017). The NLGN family includes five genes in humans (NLGN1-3, 4X, 4Y; Bolliger et al., 2001) and four genes in mice (Nlgn1-4; Bolliger et al., 2008). NLGN proteins contain an intracellular PDZ binding motif and an extracellular acetylcholinesterase domain (Levinson and El-Husseini, 2005).

Members of the NLGN family show some synapse specificity, with NLGN1 enriched at excitatory synaptic contacts, and NLGN2 at inhibitory contacts (Song et al., 1999; Varoqueaux et al., 2004; Chubykin et al., 2007). NLGN3 can be localized at either type of contact (Budreck and Scheiffele, 2007), while NLGN4 is enriched at central and peripheral inhibitory contacts (Hoon et al., 2011). This preferential enrichment pattern points to a role in synapse specificity. Indeed, Nrxn and Nlgn protein families have been shown to influence the “balance” of excitation and inhibition *in vitro* (Prange et al., 2004; Levinson and El-Husseini, 2005), and in animal models (Hines et al., 2008; Dahlhaus et al., 2010; Pizzarelli and Cherubini, 2011). Specific genes within NRXN and NLGN families of CAMs have been implicated in the etiology of ASD, including: NLGN1, NLGN2, NLGN3, NLGN4, NRXN1, NRXN2, CNTNAP2, and CNTNAP4 (Jamain et al., 2003; Banerjee et al., 2014; Baig et al., 2017). Alterations to NRXN and NLGN family members in ASD range from *de novo* mutations to common genetic variants, and include polymorphisms, variants, frameshift mutations, truncations, and deletions. Although validated in multiple independent studies these mutations are relatively rare (Jamain et al., 2003; Hedges et al., 2012; Banerjee et al., 2014). CNTNAP2 (Arking et al., 2008; Anney et al., 2012) and NRXN1 (Sanders et al., 2011) in particular have been validated through genome-wide association studies.

Several animal models have been developed to examine the contributions of Nrxn to excitatory signaling and the symptoms of ASD (Cattabeni et al., 1999; Kattenstroth et al., 2004; Grayton et al., 2013; Dachtler et al., 2014) but information about inhibitory signaling in these models is limited. Ablation of individual *Nlgn* genes in mouse models have somewhat subtle effects on development and behavior likely due to compensation, yet combined deletion of *Nlgn*1-*3* results brainstem inhibitory dysfunction leading to respiratory failure (Varoqueaux et al., 2006). Detailed examination of Nlgn1 KO mice revealed an impairment in *N*-methyl-D-aspartate (NMDA) signaling consistent with enrichment at excitatory synapses, along with repetitive behavior and impaired spatial memory (Blundell et al., 2010). Nlgn3 KO mice show a lack of social novelty preference and reduced vocalization, accompanied by an olfactory deficit (Radyushkin et al., 2009).

Expression of one of the Nlgn3 mutations identified in autism (R451C substitution) was shown to result in impaired social interactions paralleled by an increase in inhibitory synaptic transmission, leaving excitatory synapses unaffected (Tabuchi et al., 2007). However, a follow up study was not able to reproduce the deficits in social interaction in Nlgn3R451C mice (Chadman et al., 2008). In these studies Nlgn3R451C mice were shown to have impairments in ultrasonic vocalizations, and other subtle indications of impaired development (Chadman et al., 2008). In another follow up study the Nlgn3R451C mutation was backcrossed onto a different background strain (129S2/SvPasCrl), which reproduced deficits in social interaction (Jaramillo et al., 2014). The discrepancies among these studies may be attributed to subtle differences in methodology, particularly given that the social interaction tasks in mice are very sensitive to variations in protocol. Nlgn4 loss of function mutation results in deficits in reciprocal social interaction and vocalizations (Jamain et al., 2008).

Increased expression of NLGNs has also been shown to result in several abnormalities relevant to ASD, including behavioral deficits. Increased expression of Nlgns1-4 was shown to result from KO of the eukaryotic translation initiation factor 4E-binding protein 2 (4E-BP2; Gkogkas et al., 2013). 4E-BP2 is a repressor of eukaryotic translation initiation factor 4E (eIF4E), which is downstream in the signaling cascade of mammalian target of rapamycin (mTOR). 4E-BP2 KO mice exhibit an altered ratio of excitatory to inhibitory synapses, along with deficits in social interaction, ultrasonic vocalizations, and the occurrence of repetitive behaviors (Gkogkas et al., 2013). In other studies, transgenic mice with an overexpression of Nlgn1 and Nlgn2 were generated to determine the effect of specific single Nlgns on the excitatory to inhibitory ratio (Hines et al., 2008; Dahlhaus et al., 2010). Mice with enhanced expression of Nlgn1 show deficits in memory acquisition, along with enlarged excitatory synapse morphology and enhanced excitatory signaling (Dahlhaus et al., 2010). Mice with the enhanced expression of Nlgn2 were found to have behavioral symptoms of neurological disorders similar to those seen in RS (limb clasping), decreased social interaction, repetitive behaviors, as well as decreased viability. Immunohistochemical, electron microscopic and electrophysiological analysis of the Nlgn2 overexpressing mice revealed a shift in the excitatory to inhibitory ratio toward inhibition, with an increase in inhibitory synaptic contacts and overall enhancement of inhibitory responses (Hines et al., 2008). Nlgn2 has been shown to directly interact with gephyrin, which is known to aid in localizing and stabilizing GABA_A_Rs (Pizzarelli and Cherubini, 2011). Of particular interest, a recent study has shown that the NLGN2 R705C mutation identified in autism disrupts the interaction with gephyrin and thus has consequences for GABA_A_R function (Nguyen et al., 2016).

Although associated with contactins (discussed below), CNTNAP proteins are members of the NRXN family based on structural similarity, and are also associated with ASD (Katidou et al., 2008; Burbach and van der Zwaag, 2009). CNTNAP genes code for CASPR proteins which act as CAMs. Similar to NRXNs, CASPR proteins contain LNS domains, and EGF-like repeats, but also contain discoidin homology domains, and an extracellular fibrinogen-like domain (Katidou et al., 2008; Burbach and van der Zwaag, 2009). Within the CASPR cytoplasmic tail is a protein 4.1 biding site and class II PDZ binding motif (Katidou et al., 2008; Burbach and van der Zwaag, 2009).

Genetic analysis of CNTNAP2 in ASD has revealed deletions producing a frameshift mutations leading to early introduction of a stop codon (Strauss et al., 2006; Watson et al., 2014). This causes a complete loss of function in the CASPR2 protein in homozygous individuals. Following this implication of CNTNAP2, several studies have shown a link between CNTNAP2 and ASD risk (Alarcón et al., 2008; Arking et al., 2008; Bakkaloglu et al., 2008; Vernes et al., 2008). In human populations, CNTNAP2 heterozygous mutations do not always produce symptoms of ASD, while homozygous individuals for the mutation display ASD symptoms, along with epilepsy, facial dysmorphisms, severe intellectual disability and impaired language (Rodenas-Cuadrado et al., 2016). CNTNAP2 has been shown to cluster voltage gated potassium channels at nodes of Ranvier (Poliak et al., 1999), and influence dendritic arborization, synapse strength, and plasticity (Anderson et al., 2012). In mice, homozygous KO of CNTNAP2 causes increased grooming and digging (repetitive behaviors), impaired ultrasonic vocalization, less time interacting, and epilepsy (Peñagarikano et al., 2011). The behavioral changes in CNTNAP2 KO mice are paralleled by a decrease in PV-positive interneurons, suggesting that CNTNAP2 plays role in GABAergic interneuron development (Peñagarikano et al., 2011).

Both *de novo* and maternally inherited deletions have been detected in ASD populations in a related family member, CNTNAP4 (O’Roak et al., 2012). CASPR4 protein is enriched in the presynaptic terminals of developing interneurons, and CNTNAP4 KO mice show a reduction in GABAergic signaling from PV-positive interneurons in the cortex (Karayannis et al., 2014). CNTNAP4 KO mice were also found to have enhanced midbrain dopamine release into the nucleus accumbens, paralleled by increased responsiveness to startle and enhanced sensory motor gating (linked to schizophrenia), along with excessive grooming behavior (Karayannis et al., 2014). Treatment of CNTNAP4 KO mice with an α1-selective positive allosteric modulator at GABA_A_Rs, indiplon, ameliorated the sensory-motor gating phenotype (Karayannis et al., 2014).

CASPR proteins interact with the contactin (CNTN) family of proteins. CNTN proteins perform diverse functions in the CNS including myelination, synapse formation, and plasticity. This group of proteins, like the NRXNs and NLGNs, is also adhered to the cell membrane (Zuko et al., 2013). Duplication or deletion mutations in CNTN4 near CNTN3 have been implicated in the development of ASD, with more rare mutations in CNTN5 and 6 also being implicated (Banerjee et al., 2014). CNTN4 plays a role in the growth and development of axons and maintenance of mature networks. In contrast to CNTNAP mutations, loss of a single functional copy of CNTN4 can cause developmental delays (Roohi et al., 2009).

### Scaffolding Proteins: SHANK Family of Proteins

Mutations in genes encoding scaffolding proteins, such as the SHANK family of proteins have also shown connection to the development of ASD (**Table 2**; Leblond et al., 2012; Sato et al., 2012; Monteiro and Feng, 2017). Scaffolding proteins function in clustering and anchoring synaptic proteins including neurotransmitter receptors, and serve to link post synaptic receptors with downstream signaling components. The SHANK family includes three genes (SHANK1-3), that can be alternatively spliced to generate multiple Shank protein variants (Monteiro and Feng, 2017). Full-length Shank proteins are composed of 5–6 N-terminal ankyrin repeat domains, a Src Homology 3 (SH3) domain, a PDZ domain, a proline-rich region and a C-terminal sterile alpha motif (SAM) domain (Monteiro and Feng, 2017). Based on the interactions of these domains, Shank can bind an estimated 30 synaptic partners, including other scaffold molecules, glutamatergic receptors, and cytoskeletal proteins (Ehlers, 1999; Sheng and Kim, 2000; Baron et al., 2006; Gundelfinger et al., 2006). Shank proteins are expressed in the postsynaptic compartment of glutamatergic synapses. As central regulators of receptors, signaling machinery, and the cytoskeleton at excitatory synapses, Shank proteins have been shown to be involved in spine morphogenesis, synapse formation, and glutamate receptor trafficking (Naisbitt et al., 1999; Sala et al., 2001, 2005; Gerrow et al., 2006; Verpelli et al., 2011).

Mutations in all three SHANK family members have been connected with ASD and/or associated disorders (Ting et al., 2012; Leblond et al., 2014). SHANK1 deletions have been detected in ASD males (Sato et al., 2012), while multiple studies have detected *de novo* mutations and deletions in SHANK2 (Berkel et al., 2010; Pinto et al., 2010; Leblond et al., 2012). SHANK3 is even more strongly implicated in ASD and related disorders. SHANK3 haploinsufficiency is responsible for Phelan McDermid syndrome, which is characterized by intellectual disability, hypotonia, epilepsy, and ASD characteristics (Phelan and McDermid, 2012). In varied ASD populations, *de novo* mutations, truncations and terminal deletions have been detected in SHANK3 (Durand et al., 2007; Boccuto et al., 2013; Leblond et al., 2014). SHANK3 has also been identified in genome wide association studies of ASD (Connolly et al., 2017).

Modeling of these mutations in mice has revealed that many of the mutant proteins do not achieve their synaptic localization, and are retained in the cell body or nucleus (Grabrucker et al., 2014), and that a variety of SHANK manipulations *in vivo* can produce ASD relevant phenotypes (Sala et al., 2015). SHANK2 mutations in mice result in upregulation of glutamate receptors and a decrease in synaptic transmission (Schmeisser et al., 2012). Behaviorally, SHANK2 mutant mice are hyperactive, exhibit repetitive grooming, and have impairments in social interactions and vocalizations (Schmeisser et al., 2012). SHANK3 KO leads to reduction in spine volume, a decrease in the thickness of the postsynaptic density, and a loss of dendritic spines (McGee et al., 2014). SHANK3 KO mice show abnormal social behaviors, and impairments in learning and memory (Bozdagi et al., 2010). SHANK3 mutations identified in patients with ASD show a modification in dendritic spine induction and morphology as well as actin accumulation in spines affecting growth cone motility which is nicely paralleled by the mouse model (Durand et al., 2007). SHANK3 duplication in mice leads to hyperactivity and spontaneous seizures much like human subjects who have small duplications in the SHANK3 locus (Han et al., 2013). KO mice for SHANK3 showed abnormal communication patterns, repetitive behaviors, and impaired learning (Banerjee et al., 2014; McGee et al., 2014). Again, these behaviors are consistent with the phenotype of ASD in humans.

SHANK3 interacts with NLGN genes as a binding partner to play role in synaptic plasticity (Meyer et al., 2004), and SHANK3 is also involved in synapse formation and this results in an increase of excitatory signals (Arons et al., 2012). Although the link of Shank proteins to excitatory synapse function is clear, studies have not thoroughly investigated the impact that SHANK mutations may have on interneuron or inhibitory synapse function, downstream of excitation. Further studies on the existing SHANK mouse models generated may be helpful in examining the relationship between excitatory synapse dysfunction and downstream impacts on inhibitory signaling.

### 15q11-q13 Locus Genes: GABA_A_ Receptor Subunits

A known unstable genetic locus, termed 15q11-q13, is located on chromosome 15 and contains UBE3A (Ubiquitin-protein ligase E3A), and three GABA_A_R subunit genes (*GABRB3*, *GABRA5*, and *GABRG3*; **Table 2**). The 15q11-q13 region is subject to imprinting and recombination, and is regarded as one of the most complex regions in the genome (Martin et al., 2000). The partial trisomy of chromosome 15 was first reported as a syndrome in 1975 (Hecht, 1975). The syndrome produced by duplication of 15q11-q13, termed Dup15q, includes global developmental delay, social communication deficits, intellectual disability, with a high incidence of epilepsy and/or infantile spasms (Urraca et al., 2013). Children with Dup15q generally meet the diagnostic criteria for ASD diagnosis (Urraca et al., 2013), and several studies have now shown that duplications in 15q11-q13 are one of the most common copy number variants linked with ASD, which have been replicated in genome wide association studies (Cook et al., 1997; Park and Bolton, 2001; Kwasnicka-Crawford et al., 2007; Depienne et al., 2009; Sanders et al., 2011). Duplications in this region can take one of two forms, two extra copies of the 15q11.2-q13.1 region of maternal origin on a supernumerary chromosome (isodicentric), or one or more extra copies on the q arm of chromosome 15 (DiStefano et al., 2016). 15q11-q13 loss of activity is discussed specifically below in the “Angelman Syndrome” section.

Duplication specifically of the region containing GABA_A_Rs leads to a prediction of excessive inhibitory neurotransmission due to gene dosage; however, an *in vitro* model of 15q duplication displayed reduced transcripts for GABRB3 (and other nearby genes) due to impaired homologous pairing (Meguro-Horike et al., 2011). The symptoms of individuals with Dup15q also are suggestive of altered GABAergic control of oscillatory activity. Multiple reports have demonstrated enhanced activity in β frequency bands (12–30 Hz) of the EEG, with spontaneous β oscillations visually evident (Urraca et al., 2013). A further study demonstrated that enhanced power in the upper β frequencies (20–30 Hz) was significantly related to epilepsy diagnosis in Dup15q (Frohlich et al., 2016). The enhanced power and spontaneous oscillations noted in Dup15q are similar to the effects seen following treatment with benzodiazepines and other allosteric modulators of GABA_A_Rs, although none of the patients were on such medication (Urraca et al., 2013; Al Ageeli et al., 2014; Frohlich et al., 2016). Further, since the EEG β phenotype is observed with both maternal and paternal duplication cases, it is likely that this phenotype results from altered GABA_A_R function and not the maternally expressed (non-imprinted) Ube3a gene (Frohlich et al., 2016).

Examination of the seizure phenotype in patients with Dup15q have indicated about 63% of those with isodicentric mutations have seizures, with the majority of these cases having multiple seizure types (81%), and with a relatively high rate of infantile spasms (42%; Conant et al., 2014). In contrast, only about 25% of interstitial mutation type Dup15q patients had seizures (Conant et al., 2014). This study also revealed that broad spectrum anti-epileptic drugs, and those that block voltage gated sodium channels (carbamazepine and oxcarbazepine) were typically effective, whereas typical benzodiazepines were relatively ineffective (Conant et al., 2014). Also indicative of abnormal GABAergic signaling, paradoxical increases in seizure severity have been reported in isodicentric Dup15q following treatment with pregabalin (Di Rocco et al., 2013), a drug that acts to increase extracellular GABA by increasing the density of GABA transporter proteins, increasing the rate of functional GABA transport, and increasing the enzyme responsible for making GABA (L-glutamic acid decarboxylase) as a result of high-affinity binding to the α2-δ subunit of voltage-gated calcium channels (Sze, 1979; Marks et al., 2009). Mouse models mimicking human 15q11-q13 duplication exhibit features of autism, such as poor social interaction, behavioral inflexibility, and abnormal ultrasonic vocalizations (Nakatani et al., 2009). Further, a mouse model with a point mutation altering the surface stability of the GABA_A_R β3 subunit (GABRB3 gene product) displays ASD-like features and enhanced susceptibility to seizure (Vien et al., 2015).

Indirectly reacted to the findings of loss of function of GABA_A_R in Dup15q, postmortem studies from subjects with ASD with non-specified genetic background have shown reduced levels of [^3^H]-muscimol binding compared to control (Blatt et al., 2001). Muscimol has a higher affinity for α4/δ-containing receptors than other GABA_A_R (Huh et al., 1996). Recently, expression and protein levels of subunits where found to be significantly reduced in different regions of brains from autistic subjects compared to age-matched controls. Protein expression of α1, α2, α3, α5, and β3 subunits were reduced in the parietal cortex whereas in the frontal cortex α1, α4, α5, and β1 subunits were reduced (Fatemi et al., 2009, 2010, 2013). These data would suggest a GABAergic hypofunction and in particular, a reduced tonic inhibition of patients with autism.

Other rare mutations implicated in ASD are directly or indirectly linked to GABAergic signaling, including, mutations in SLC12A5 impacting the C-terminal regulatory domain of KCC2 (Merner et al., 2015), *RIM1A* (Iossifov et al., 2014) which has been shown to modulate GABA release from interneurons (Lachamp et al., 2009), *DLX* homeobox transcription factors (International Molecular Genetic Study of Autism Consortium [IMGSAC], 2001; Liu et al., 2009) which play a role in interneuron differentiation and induction of GABA synthesis (Pleasure et al., 2000; Cobos et al., 2005; Paina et al., 2011), and *ANK2* (Ankyrin B; De Rubeis et al., 2014; Iossifov et al., 2014) which creates the AIS boundary, and is involved in modifying AIS length (Galiano et al., 2012). Collectively, these genetic and animal model studies suggest that dysfunction in the structure and or function of transmembrane adhesion, scaffolding, and receptor proteins critical for creating and stabilizing synapses are major contributors to ASD. In particular, evidence of altered inhibitory synapse adhesion molecules, scaffolding proteins, and GABA_A_R signaling is clearly implicated in ASD. Alterations in PV-positive neuron number and/or function, and alterations in tonic inhibition are common among multiple genetic causes of ASD, warranting further study. Below we will explore monogenetic NDDs with symptoms related to ASD, to examine the indications of inhibitory synapse dysfunction more broadly.

## Angelman Syndrome

Angelman syndrome (AS) is a NDD characterized by severe mental retardation, ataxic movements, speech impairment, and high incidence of seizures (Buiting et al., 2016). In contrast to other NDDs, people with AS display a sociable disposition and are prone to unprovoked bouts of laughter. AS is relatively rare, with an incidence of somewhere between 1:12,000 and 1:20,000, accounting for about 6% of all children with mental retardation and epilepsy (Pelc et al., 2008; Buiting et al., 2016). AS is caused by a loss of activity within the 15q11-13 locus, and about 70% of cases are due to a “*de novo*” interstitial deletion in the long arm region, arising on the maternally inherited chromosome (Buiting et al., 2016). The candidate gene in this region thought to be responsible for AS is UBE3A (**Table 2**; Kishino et al., 1997; Matsuura et al., 1997; Sutcliffe et al., 1997), although three GABA_A_R subunit genes are also located in this region as mentioned above. AS is a classic example of imprinting and is most commonly caused by deletion or inactivation of genes on the maternally inherited chromosome 15 while the paternal copy is imprinted or silenced in the brain producing the most severe cases, with seizures, mental and motor retardation, dysmorphic features and microcephaly phenotype (65–70% of probands; Kyllerman, 2013). The other two possible causes, uniparental disomy and imprinting center mutations (10%), and UBE3A point mutations (11%) result in more mild phenotypes (Kyllerman, 2013). The AS diagnosis is confirmed by methylation test or by mutation analysis of the UBE3A gene. Regardless of mutation type, seizures occur between ages 1 and 3 years in approximately 85% of patients and are most commonly characterized by atypical absence, erratic myoclonic, and rare tonic–clonic seizure types (Fiumara et al., 2010). Detailed EEG studies of AS patients have revealed high-amplitude δ frequency (2–3 Hz) activity, with spike and slow-wave discharges, and a circadian pattern to epileptiform discharges, showing sleep-activation (Kyllerman, 2013).

As discussed above, the GABA_A_R genes, GABRB3, GABRA5, and GABRG3 genes (**Table 2**), encoding β3, α5, and γ3 subunits, respectively, are also within the 15q11-13 locus. The presence of this GABA_A_R subunit gene cluster in 15q11-13 has led to the hypothesis that GABA neurotransmission may also be involved in AS (Pelc et al., 2008), as well as ASD. In particular, the GABRB3 gene is known to be associated with epilepsy in humans, and when a deficiency of UBE3A is also present, more severe symptoms result (Pelc et al., 2008).

The phenotype of maternal Ube3a-deficient mice resembles human AS with motor dysfunction, inducible seizures and a context-dependent learning deficit (Jiang et al., 1998). Using mouse models it has been demonstrated that Ube3a-deficiency results in reduced GABA-mediated tonic inhibition in the cerebellum, mechanistically caused by a GAT-1 dependent decrease of GABA concentration in the extracellular space (Egawa et al., 2012). A recent paper elegantly examined the contributions of Ube3a loss in specific populations of cells to the pathophysiology of AS (Judson et al., 2016). Using conditional deletion in mouse models it was shown that loss of Ube3a in GABAergic interneurons, but not glutamatergic principal cells, results in EEG abnormalities of enhanced δ, and seizures, similar to individuals with AS. Other studies have examined the combined Ube3a and GABRB3 effects using chromosomal deletion spanning Ube3a to GABRB3 (includes a deletion of the Atp10a gene; Jiang et al., 2010). Maternal (Ube3a^m-/p++^), but not paternal (Ube3a^m+/p-^), deletion mice had increased spontaneous seizure activity and abnormal EEG, along with impairments in motor function, learning and memory, ultrasonic vocalizations, and an anxiety-like phenotype demonstrating the impact of GABA_A_R function on the severity of phenotype (Jiang et al., 2010). The Ube3a^m-/p+^ model has also been shown to have an inhibitory deficit resulting from impaired vesicle cycling in interneurons (Wallace et al., 2012). In addition to Ube3a, examination of GABA_A_R β3 subunit-deficient mouse models have also revealed a strong link between GABA_A_Rs and AS, β3 deficient mice show 90–95% neonatal mortality, with survivors displaying a phenotype resembling severe forms of human AS (Homanics et al., 1997; DeLorey et al., 1998).

The maternal deletion model of AS (Ube3a^m-/p+^) has also been shown to result in alterations to the intrinsic properties of neurons (Kaphzan et al., 2011). In these studies researchers demonstrated alterations in resting membrane potential, threshold potential, and action potential amplitude of principal cells. The changes in intrinsic properties were paralleled by significant increases in the expression of the voltage gated sodium channel NaV1.6, and the AIS anchoring protein ankyrin-G, as well as an increase in length of the AIS (Kaphzan et al., 2011). Previous studies have shown that the AIS can adapt to the inputs that a cell is receiving, with reduced synaptic input resulting in elongation of the AIS (Kuba et al., 2010), and increased excitability moving the AIS away from the cell body (Grubb and Burrone, 2010). The AIS is also of interest in considering GABAergic signaling, as it is the postsynaptic site of PV-positive chandelier cell contact, and changes in the properties of the AIS also likely effect GABAergic control at this important site.

As with ASD, AS is also clearly linked to dysfunction of inhibitory signaling, and in particular to dysfunction of GABA_A_R subunit expression and function. Again, dysfunction of PV-positive neurons emerges as a feature of AS. It is interesting that despite a common biological phenotype of reduced transcripts for GABA_A_R subunit genes in Dup15q ASD and AS, the EEG phenotype differs between these two syndromes, suggesting the need for regional, and more specific analysis of GABA_A_R subunit expression and function, as well as GABAergic synapse subtypes in these disorders.

## Rett Syndrome

Rett syndrome (RS), is a genetic disorder that leads to severe impairments, affecting nearly every aspect of a child’s life. ASD-like features, especially stereotyped behaviors, loss of language skills, learning and memory deficits, loss of social skills, and seizures are common characteristics of RS (Chahrour and Zoghbi, 2007). RS may also include apraxia, ataxia, impaired coordination, tremor, respiratory dysrhythmias, and sometimes premature lethality (Chahrour and Zoghbi, 2007; Chao et al., 2007). Not all of the symptoms of RS are present during early postnatal development, but rather appear over stages, following a period of developmental stagnation that typically begins between 6 and 18 months of age (Chahrour and Zoghbi, 2007). The primary cause of RS is mutation of the gene encoding the transcriptional repressor methyl-CpG-binding protein 2 (MeCP2; **Table 2**; Kozinetz et al., 1993). The MECP2 gene is located on the X-chromosome, and since males have only one X chromosome (instead of the two in genetic females), MECP2 mutation results in more severe symptoms, with the majority of RS males dying shortly after birth (Meloni et al., 2000). In females, RS represents one of the leading causes for mental retardation (Neul and Zoghbi, 2004). Within females, the severity of RS symptoms can vary widely depending upon the pattern of x-chromosome inactivation (Dragich et al., 2000). It is estimated that approximately 60–80% of females with RS have epilepsy (Vignoli et al., 2017), with severity of the syndrome positively correlated with the prevalence of epilepsy (Tarquinio et al., 2017). Longitudinal studies have indicated an even higher cumulative lifetime prevalence of epilepsy in RS, with approximately 90% incidence evaluated using a Kaplan–Meier method (Tarquinio et al., 2017). A fascinating link between ASD, AS, and RS was identified when researchers found that MECP2 (along with other factors) is required for the homologous pairing of 15q11-q13 during neuronal differentiation during the maturation process (Meguro-Horike et al., 2011).

Researchers have developed multiple animal models to study the underlying neuropathology associated with RS. Mecp2 deficient mice, created by deleting either exon 3, or exons 3 and 4, replicate all the features of the human disease (Chen et al., 2001). Truncation mutations (Mecp2^308^) produce a more moderate phenotype that shows the progressive pattern of symptom development (Shahbazian et al., 2002). It is also interesting to note that in mouse models (Collins et al., 2004), and in human NDDs (del Gaudio et al., 2006), increased expression of Mecp2 also produces a progressive neurological phenotype, indicating that precisely controlled levels of Mecp2 are essential for normal nervous system function.

Conditional, neuron specific deletion of Mecp2 in mice is sufficient to produce symptoms of RS (Guy et al., 2001), and the neurologic abnormalities can be reversed by activating the Mecp2 gene later in life (Amir et al., 1999; Luikenhuis et al., 2004). Surprisingly, despite the diversity of the features seen in RS, recent findings also indicate that deleting Mecp2 only from GABAergic inhibitory neurons in mice is sufficient to replicate most of the phenotypes of the human disease (Medrihan et al., 2008). In this elegantly performed study, researchers created a Mecp2 KO model by crossing Viaat-Cre to Mecp2^flox/+^ mice. The result from this study was a loss of Mecp2 in male Viaat-Mecp2^-/y^ mice from more than 90% GABAergic neurons (Chao et al., 2010). Mice with Mecp2 deficiency from GABA-releasing neurons recapitulate numerous RS and ASD features, including repetitive behaviors, ataxia, premature death, breathing abnormalities, and seizures. These mice initially exhibit normal behavior, then develop forepaw stereotyped movements, compulsive grooming, increased sociability, impaired motor coordination, learning/memory deficits, abnormal EEG hyperexcitability, severe respiratory dysrhythmias, and premature lethality (Chao et al., 2010). Specific interneuron subtypes have been shown to play distinctive roles in RS, determined by selectively deleting Mecp2 from either PV or SOM positive interneurons (Ito-Ishida et al., 2015). Deletion of MeCP2 in PV+ interneurons resulted in motor, sensory, cognitive, and social interaction deficits, whereas deletion of MeCP2 in SOM+ interneurons resulted in seizures and stereotypies (Ito-Ishida et al., 2015). Another recent study was able to genetically re-express Mecp2 solely in GABAergic neurons in a Mecp2 KO mouse, restoring inhibitory function. GABA concentrations in the striata of 8-week-old rescue mice were restored to wildtype levels. No behavioral seizures were seen in the rescue mice, while EEG recordings showed that five out of six rescue mice had normalized activity patterns (Lu et al., 2016). Another recent study demonstrated that KCC2 is a downstream target of Mecp2, and that human neurons derived from stem cells of RS patients have reduced KCC2 expression and a delayed switch in EGABA (Tang et al., 2016).

Studies examining the neuropathology of RS have identified strong depression of GABAergic neurotransmission in the ventrolateral medulla (Medrihan et al., 2008), and nucleus tractus solitarius (Chen et al., 2017) of Mecp2 null mice. Studies in the ventrolateral medulla indicated reduced GABA release paralleled by reduced levels of the vesicular inhibitory transmitter transporter and reduced levels of α2 (typically synaptic) and α4 (typically extrasynaptic) subunits of GABA_A_Rs (Medrihan et al., 2008). In the nucleus tractus solitarius, it has been shown that GABA_A_R δ subunit (typically extrasynaptic) expression is enhanced, paralleled by enhanced responsiveness to THIP, which selectively activates extrasynaptic GABA_A_R subtypes (Chen et al., 2017). Other studies have indicated reductions in the frequency of spontaneous inhibitory postsynaptic currents in hippocampal recordings from symptomatic Mecp2 null mice (Zhang et al., 2008). In addition, these studies found that Mecp2 null hippocampal circuitry was prone to hyperexcitability, as evidenced by induction of repetitive sharp wave-like discharges following a brief train of high-frequency stimulation (Zhang et al., 2008).

Similar to ASD and AS, several lines of evidence also link RS to dysfunction of inhibitory signaling. In particular, support for the role of PV-positive interneuron dysfunction in broad NDDs is apparent. In addition, indications of KCC2 dysfunction are present in both ASD and RS. Evidence is accumulating for dysfunction of specific GABA_A_R subunit expression and function, with regional specificity as well, this level of analysis is currently lacking in ASD and AS models. Studies in RS models have highlighted an interesting parameter for consideration in other NDD models, namely the ratio of synaptic to extrasynaptic GABAergic signaling (Medrihan et al., 2008; Chen et al., 2017). Changes in subunit expression and mode of inhibition (phasic versus tonic) as a direct result of mutation, or as a plastic or homeostatic adaptation could result in alterations in network function leading to some of the electrophysiological, EEG, and phenotypic manifestations of NDDs.

## Dravet Syndrome

Developmental epilepsies are also considered in the umbrella of NDD, due to the high incidence of epilepsy in prototypical NDD, as well as the developmental factors known to contribute to epileptogenesis (Bozzi et al., 2012). Further, just as epilepsy has a high occurrence in NDD populations, NDD features are also commonly observed in epilepsy syndromes. In addition to the prototypical NDDs, consideration of the phenotypes of developmental epilepsy syndromes will provide insight into the cellular and circuit dysfunction in NDD. Dravet syndrome (DS), formerly known as Severe Myoclonic Epilepsy of Infancy (SMEI), is a rare form of epilepsy that begins in the first year of life with frequent and/or prolonged seizures (Han et al., 2012). At early onset, DS is characterized by both febrile and non-febrile seizures, and progresses to myoclonic and/or partial seizures, psychomotor delay, and ataxia (Selmer et al., 2009). Patients with DS also have a high risk of sudden unexpected death in epilepsy (SUDEP; Shmuely et al., 2016). DS is also characterized by cognitive impairment, motor deficits, hyperactivity and/or impulsiveness, and in rare cases can include autistic-like behaviors (Han et al., 2012). The prevalence of DS is 1:15,700 individuals, with around 75% of patients bearing a mutation in their SCN1A gene, which encodes the α1 subunit of the voltage gated sodium channel (Na_V_1.1; Wu et al., 2015). The remaining 25% of DS cases have been linked to mutations in GABRG2, GABRA1 (**Table 2**), protocadherin 19 (PCDH 19), and Syntaxin Binding Protein-1 (STXBP1; Carvill et al., 2014). Mutations in SCN1A are also responsible for Genetic Epilepsy with Febrile Seizures Plus (GEFS+), which is an autosomal dominant form of epilepsy, typically less severe than DS (Hawkins et al., 2016; Kang and Macdonald, 2016). These findings indicate additional factors beyond SCN1A mutation that contribute to the occurrence and severity of the phenotype (Hawkins et al., 2016; Kang and Macdonald, 2016). A recent human study using single and paired pulse transcranial magnetic stimulation has found an absence of short interval intracortical inhibition in DS patients, suggestive of reduced cortical GABAergic neurotransmission (Stern et al., 2017).

Mouse models of DS (*Scn1a*^+/-^) are characterized to have spontaneous seizures, including thermally evoked seizures (Yu et al., 2006; Oakley et al., 2009), and as well as behavioral phenotypes relevant to ASD (Han et al., 2012). Electrophysiological assessment of DS models has revealed a selective loss of sodium currents in GABAergic inhibitory neurons, resulting in reduced excitability of interneurons and thus reduced GABAergic neurotransmission (Yu et al., 2006; Kalume et al., 2007; Cheah et al., 2012; Han et al., 2012). Field recordings in Scn1A^+/-^ mice have demonstrated hyperexcitability of hippocampal circuits in both the pre-epileptic and epileptic periods (Liautard et al., 2013). Similar to the human variation in epilepsy severity, mouse models of SCN1A dysfunction, have been shown to have a strain dependent epilepsy phenotype, raising the possibility of additional factors contributing to pathogenesis (Hawkins et al., 2016). Scn1a^+/-^ mice on 129 background have no readily detectable phenotype and a normal lifespan, while Scn1a^+/-^ F1 129/C57Bl6 hybrid mice experience spontaneous and hyperthermia-induced seizures and high rates of premature death. Genetic mapping comparing Scn1A^+/-^ mutations on 129 versus F1 hybrid backgrounds identified multiple modifier loci, followed by RNA-Seq of a loci of interest on chromosome 5 (Hawkins et al., 2016). RNA-Seq revealed three genes with significant differences in total gene expression between Scn1A deletion on the two backgrounds, including the α2 subunit of GABA_A_Rs, associating low *Gabra2* expression with the B6 allele and reduced survival of Scn1A mutants on this background (Hawkins et al., 2016). These studies also showed that clobazam, which has preferential affinity for the GABA_A_R α2 subunit, normalized the temperature threshold for hyperthermia-induced seizures in *Scn1a*^+/-^ mice (Hawkins et al., 2016). Compared to wildtype mice, Scn1a^+/-^ mice displayed behavioral/social deficits as shown by circling behavior and less time spent in open arms in an elevated plus maze. These stereotypical behaviors are marked as autistic traits, indicating that DS does have shared symptomatology (Han et al., 2012). PV-positive and SOM-positive fast-spiking interneurons have also been found to have reduced excitability in Scn1a^+/-^ mice, leading to reduced GABAergic inhibition (Tai et al., 2014). Greater understanding of Scn1a localization and function may also reveal insights about pathogenesis in DS. The protein encoded by Scn1a, the α1 subunit of Na_V_1.1, has been localized to the AIS, particularly in GABAergic interneurons (Duflocq et al., 2008; Lorincz and Nusser, 2008). AIS dysfunction is a proposed mechanism of neuropathology in epilepsy (Wimmer et al., 2010; Kole and Stuart, 2012), and chandelier cell cartridges opposed to α2 containing GABA_A_Rs are known to mediate inhibitory control at this site (Nusser et al., 1996; Freund and Katona, 2007; Gao and Heldt, 2016), suggesting that GABAergic dysfunction at the AIS may be a key factor in epilepsy. Of particular interest, studies have also shown that the benzodiazepine clonazepam is effective at reducing the ASD-like features of the Scn1a^+/-^ mice (Han et al., 2012).

Examination of developmental epilepsies, alongside NDDs with epilepsy as an associated symptom is useful in elucidating common mechanisms. *De novo* mutation of *SCN2A* was also recently identified using exome sequencing of ASD samples (De Rubeis et al., 2014; Iossifov et al., 2014), further suggesting that the AIS may be a key site to evaluate in NDDs. As we have seen with NDD, developmental epilepsy syndromes such as DS also highlight GABAergic dysfunction as a key pathogenic mechanism. Again a common indication of PV-positive interneuron dysfunction is revealed, drawing commonality with ASD, AS, and RS. Greater understanding of the specific roles of interneuron subtypes, subcellular domains of inhibition, and GABA_A_R subunits in these disorders may help to further advance our understanding, and improve therapeutic strategies in NDDs.

## Very Rare Mutations and Associated Disorders

Insight into NDDs may also be gleaned by examining rare genetic causes and disorders. Of particular interest, mutations in the *ARHGEF9* gene (**Table 2**) have been reported to result in NDDs with varied behavioral symptoms (Alber et al., 2017). The *ARHGEF9* gene codes for the GDP/GTP exchange factor collybistin (also known as HPEM2 in humans), which is a neuron specific RhoGEF. All collybistin isoforms possess three major functional domains: an N-terminal SH3 domain, a GEF domain, and a PH domain. Through these domains, collybistin has been shown to interact with gephyrin, neuroligin-2, and the GABA_A_R α2 subunit, playing an important role in clustering of GABA_A_Rs at inhibitory synapses (Saiepour et al., 2010). To this point, 18 patients have been described to have either point mutations, chromosomal rearrangements and deletions involving *ARHGEF9*, with a range of manifestations including ASD (Bhat et al., 2016; Machado et al., 2016), behavior disorders (ADHD, anxiety, aggression; Kalscheuer et al., 2009; Lesca et al., 2011), intellectual disability (Kalscheuer et al., 2009; Lesca et al., 2011; Marco et al., 2011; Shimojima et al., 2011; de Ligt et al., 2012; Lemke et al., 2012), hyperekplexia (Harvey et al., 2004; Marco et al., 2008), and infantile epilepsy (Harvey et al., 2004; Kalscheuer et al., 2009; Lesca et al., 2011; Shimojima et al., 2011). The majority of patients identified are male, and those that are female have been shown to have a near complete X-inactivation in favor of the affected gene (Marco et al., 2008; Kalscheuer et al., 2009). Collybistin KO mice recapitulate some aspects of the behavioral syndrome including anxiety-like behavior, learning deficits, and seizures (Papadopoulos et al., 2007; Papadopoulos and Soykan, 2011). Related to the collybistin mutations, rare mutations have also been identified in gephyrin associated with diverse NDD diagnoses (**Table 2**), including ASD, seizures, and schizophrenia (Lionel et al., 2013). Gephyrin KO mice die shortly after birth, and exhibit a phenotype of tactile-hyperresponsivity and difficulty breathing consistent with glycinergic signaling deficits (Feng et al., 1998). In general, further information is required to understand the detailed contribution of collybistin, gephyrin and other inhibitory synapse organizers in specialized subtypes of GABAergic synapses, and NDD. For both collybistin and gephyrin, studying the effects of the human mutations in mouse models may yield more clarity into the role of these proteins in NDD. Other rare mutations implicated in NDDs are indirectly linked to GABAergic scaffolding and signaling, including *NONO* (Mircsof et al., 2015; Reinstein et al., 2016) which impacts the expression of both gephyrin and the GABA_A_R α2 subunit (Mircsof et al., 2015). The data from rare NDD syndromes solidify direct and indirect links between NDDs and the proteins responsible for inhibitory GABA_A_R signaling.

## Discussion

Throughout this review we have identified commonalities in GABAergic signaling dysfunction in diverse NDDs. Specific perturbation in GABAergic signaling are emerging as ribs of commonality in these diverse disorders. One emerging rib centers on perturbations in the developmental excitatory to inhibitory shift in EGABA, caused by the onset of KCC2 expression. Studies have now directly indicated SLC12A5 gene (KCC2) mutation in ASD, with the C-terminus being impacted (Merner et al., 2015). Recent examination of the molecular structure of KCC2 reveals the C-terminus to be important for dimerization and function (Agez et al., 2017). Modeling of ASD and RS associated mutations has also revealed a delay in the chloride reversal associated with alterations in KCC2 expression and function, producing corresponding alterations in neuronal signaling (Tang et al., 2016; Amin et al., 2017). Environmental models of ASD, including maternal immune activation and valproic acid exposure have also indicated reductions in KCC2 expression and delays in the shift to inhibitory GABA (Corradini et al., 2017; Li et al., 2017). Clinical Trials of bumetanide, a high affinity inhibitor of NKCC1, have been successful in reducing the core symptoms of ASD (Lemonnier et al., 2012, 2017). A second emerging rib is the control of excitation by PV-positive cells. PV positive cells contact sites at or near action potential generation in neurons, exerting powerful control over excitatory firing patterns (**Figure 1**). A number of studies have indicated perturbations in PV-positive cells in NDD populations and mouse models (Peñagarikano et al., 2011; Karayannis et al., 2014; Hashemi et al., 2017; Soghomonian et al., 2017; Vogt et al., 2017; Ariza et al., 2018). While several studies have indicated a loss of PV-positive cells, some evidence suggests that it may be a reduction in PV expression, bringing cells below the detection threshold (Filice et al., 2016). These studies are supported by the demonstration that knockout of PV in the mouse leads to behavioral phenotypes relevant to human ASD (Wöhr et al., 2015). A third emerging rib is the role of tonic inhibition (**Figure 2**, left), early in development and in NDDs. Studies have identified alterations in the expression of tonic GABA_A_R subunits in human populations (Fatemi et al., 2014), as well as deficits in tonic inhibition in mouse models of NDD (Olmos-Serrano et al., 2011; Egawa et al., 2012; Martin et al., 2014; Vien et al., 2015; Zhong et al., 2015; Bridi et al., 2017). Gaboxadol, a GABA_A_R agonist with preferential activity at δ-containing receptors, is being examined in clinical trials for patients with Fragile-x and AS (Ligsay et al., 2017).

Despite these emerging ribs, our understanding of the details of inhibitory signaling lags behind that which is known about excitatory signaling. In particular, further investigation of regional or subtype specific deficits in GABAergic signaling is warranted in in NDD populations and animal models. More specific information about GABA_A_R subunit alterations would provide insights about which modes of inhibition, and which subtypes of inhibitory synapses may be contributing to NDDs. Investigations comparing both phasic and tonic forms of inhibition across animal models of NDD would be illuminating, as tonic inhibition plays an important and powerful role in controlling brain activity but is understudied. In addition, subtypes of synaptic GABA_A_Rs, including somatic and AIS synapses, are known to have a powerful role in coordinating neuronal activity patterns, but we are just beginning to understand the roles of these synapses, how they are specified, how they develop, and their possible contributions to NDDs. In addition, there are many key adhesion, and scaffolding proteins required for the aforementioned processes at specific inhibitory synapse subtypes, including neuroligins, dystroglycan, dystrophin, gephyrin, and collybistin, among others. Increasing our understanding of these key proteins involved in the formation, maintenance, and function of specific inhibitory synapses will also advance our understanding of how subtypes impact neurodevelopment more globally.

Further, assessments of specific interneuron function also seem promising, with evidence pointing toward PV-positive interneuron dysfunction as a key factor mentioned above. In particular, it may be important to examine the mechanisms causing changes in PV expression, as well as understand the functional implications of such changes to neuronal circuit activity. In addition, analysis of markers for other subtypes of interneurons in NDD and mouse models have not been described. In particular, analysis of interneuron targeting interneurons that have disinhibiting effects on network signaling is lacking in NDD populations and models. This is of particular relevance as a recent study has shown that Nav1.2 (SCN2A), which bears an ASD related mutation (De Rubeis et al., 2014), is enriched in inhibitory cells that innervate other interneurons (Yamagata et al., 2017). Analysis of circuit level dysfunction in NDD populations and models is also currently focused on seizures and epilepsy, and more global assessment of patterned oscillatory activity may provide the next needed advance following specific inhibitory mode, interneuron, and synapse subtype assessments. In particular, systematic assessment of oscillatory activity across multiple mouse models of NDDs may provide illuminating insights into commonalities and distinctions. Also of importance is to evaluate and/or manipulate circuit activity during execution of specific behaviors relevant to NDD, allowing for further insight into the mechanisms producing symptoms, and promoting novel therapeutic approaches by modulating specific circuits to correct perturbations in oscillatory activity. Advances in our understanding of GABAergic signaling in normal development and in NDD models and populations will ultimately advance our therapeutic strategies, improving patient outcomes.

## Author Contributions

RAR contributed to the writing. CJ contributed to the writing and tables. RH conceived of the topic, wrote the manuscript, and prepared the figures.

## Conflict of Interest Statement

The authors declare that the research was conducted in the absence of any commercial or financial relationships that could be construed as a potential conflict of interest.
